# Tilting the Scale: Current Provider Perspectives and Practices on Breastfeeding with HIV in the United States

**DOI:** 10.1089/apc.2022.0178

**Published:** 2023-02-13

**Authors:** Allison Lai, Elisabeth S. Young, Hannah Kohrman, Gabriela Chateau, Deborah Cohan, Lealah Pollock, Monica Hahn, Barbara Namusaazi, Ornella Tankeu Toini, Judy Levison, Theodore Ruel

**Affiliations:** ^1^ Department of Pediatrics, University of California San Francisco, San Francisco, California, USA.; ^2^ Department of Pediatric Hospital Based Medicine, Ann & Robert H. Lurie Children's Hospital of Chicago, Chicago, Illinois, USA.; ^3^ Department of Pediatrics, Northwestern University Feinberg School of Medicine, Pediatrics, Chicago, Illinois, USA.; ^4^ Department of Neonatal Intensive Care Nursery, University of California San Francisco, San Francisco, California, USA.; ^5^ Department of Pediatric Intensive Care, Lucile Packard Children's Hospital at Stanford Pediatrics, Palo Alto, California, USA.; ^6^ Department of Obstetrics and Gynecology, University of California San Francisco, San Francisco, California, USA.; ^7^ Department of Family and Community Medicine, University of California San Francisco, San Francisco, California, USA.; ^8^ Community Advisor, Department of Pediatrics, University of California San Francisco, San Francisco, California, USA.; ^9^ Department of Obstetrics and Gynecology, Baylor College of Medicine, Houston, Texas, USA.; ^10^ Department of Pediatric Infectious Disease, University of California San Francisco, San Francisco, California, USA.

**Keywords:** breastfeeding, breastfeeding with HIV, HIV, high-income countries, national guidelines

## Abstract

The risk of vertical transmission from breastfeeding with HIV (BFHIV) has been found to be very low in optimal scenarios with sustained maternal viral suppression during pregnancy and postpartum. Medical providers must account for the risk of this serious adverse event alongside parental autonomy, breastfeeding benefits, and patient values. To assess provider practices, comfort, and challenges with BFHIV, an online mixed-method survey was sent to breastfeeding and HIV provider listservs from June to July 2021. The target population was US medical professionals from diverse practice settings with experience in clinical issues associated with BFHIV, including physicians, advanced practice providers, nurses, and lactation consultants. Data analysis utilized nonparametric hypothesis testing, ordinal regression, and reflexive thematic analysis. Most providers reported counseling pregnant people with HIV on infant feeding choices, but fewer specifically endorsed counseling about breastfeeding. Of 84 unique institutions identified by 100 included respondents, 10% had an institutional protocol supporting BFHIV. Institutional protocols were associated with higher degrees of provider comfort with BFHIV in optimal scenario clinical vignettes. Providers perceived that White patients faced fewer BFHIV barriers than patients with other racial identities. Discomfort balancing the goals to protect infants from infection risk and support the parent's role in infant feeding decisions was a key theme in free text responses; this manifested in a spectrum of management styles ranging from patient's informed choice to paternalism. This study highlights the tension providers navigate regarding BFHIV discussions, calling for patient care guidelines and protocols grounded in risk reduction and respect of patient autonomy.

## Introduction

Breastfeeding with HIV (BFHIV) in high-income countries is a charged issue with medical and ethical complexity. Medical guidelines regarding BFHIV must balance the desires to eliminate risk of HIV transmission and to uphold parental autonomy.^1^ Historically, United States (US) guidelines have recommended strict avoidance of BFHIV,^2,3^ aiming to minimize risk of vertical transmission. In contrast, World Health Organization guidelines have supported exclusive BFHIV, recognizing the high risk of mortality in settings with limited access to formula and unsafe drinking water.^4^ However, in light of mounting data about the low risk of transmission from breastfeeding in the setting of viral suppression, there is increasing interest in supporting BFHIV in high-income countries.^5^

Many studies have shown that treating pregnant and lactating people with antiretroviral therapy reduces the risk of vertical transmission^6,7^ and the landmark Promoting Maternal Infant Survival Everywhere (PROMISE) trial demonstrated that very low absolute risks of transmission from BFHIV could be achieved in the setting of maternal viral suppression. The PROMISE trial randomized 2400 mother-infant dyads with high maternal CD4 counts to either maternal antiretroviral therapy or infant prophylaxis throughout breastfeeding in sub-Saharan Africa and India.

The overall probability of vertical transmission at 6 and 12 months of age was 0.3% and 0.6%, respectively (with no difference between the arms).^8^ There were two cases of postpartum HIV transmission in the maternal antiretroviral therapy arm, despite undetectable maternal viral loads at the time of the infant positive test.^9^ This very low risk of HIV transmission while breastfeeding, along with the known benefits of breastfeeding, has led parents and providers to advocate for increased support for parents living with HIV to make informed infant-feeding decisions.^5^

Many factors influence a person/people living with HIV's (PLHIV) decision to breastfeed, including sociocultural factors, personal values, desire for infant bonding, prioritization of bodily autonomy, and stigma.^10,11^ In addition, given national formula shortages, economic strain in the setting of the COVID pandemic,^12^ and areas with unsafe water sources in the US,^13^ what was thought to be ubiquitous access to formula in the US may not be the reality for all. Breastfeeding has also been linked to improved performance on intelligence tests,^14^ lower mortality from infectious diseases, and decreased childhood or adulthood overweight/obesity prevalence.^15^ Moreover, breastfeeding may mitigate many infant and maternal conditions (e.g., asthma, type 2 diabetes, respiratory infections)^16^ that disproportionately impact Black, Indigenous, and People of Color,^17,18^ who are also disproportionately impacted by HIV.^19^ Given these considerations, a consistently undetectable viral load throughout pregnancy and postpartum represents a clinical equipoise when it comes to infant feeding.^16,20^

These emerging perspectives support BFHIV as ethically justifiable and even preferable in some scenarios.^10^ Over time, US guidelines have evolved to include more harm and risk reduction language.^21–23^ Given the discordance of perspectives on whether or not PLHIV should be supported with breastfeeding in the US, we sought to describe how providers nationally navigate infant feeding with PLHIV. This study utilizes a mixed methods national survey to describe US medical provider practice, comfort, and perceived challenges relating to BFHIV.

## Methods

### Study design, population, and data collection

We created an online survey with thirty multiple choice and eight free response questions. The survey allowed respondents to skip questions, choose not to disclose demographic information, select multiple answer choices for specified questions, and only receive certain relevant questions when they had experience with BFHIV due to branching logic. Before dissemination, the survey was reviewed by two community advisors in individualized focus groups and piloted with six public health and medical professionals; all input was incorporated into survey design. Nine questions focused on demographics, four on formal and informal institutional practice, two on individual practice, ten on provider comfort and perceptions, four on equity and ethics, seven on prior experience and outcomes, and two on survey feedback. Survey respondents ranked their comfort in optimal or outside of optimal scenarios with four clinical situations (Table 1). The finalized survey was built into an online platform for distribution (Qualtrics, Provo, UT).

**Table 1.** Clinical Situations Used to Assess Provider Comfort

	“Rate your level of comfort with the following …”^a^
General comfort in optimal scenario	“I am comfortable with supporting a patient to BFHIV in ideal conditions (e.g., shared decision making, informed consent, proper counseling, agreement to medication adherence, and undetectable viral load maintained through pregnancy).”
General comfort outside of optimal scenario	“I am comfortable supporting a patient who chooses to BFHIV despite counseling, who does not fit these ideal conditions (e.g., shared decision making, informed consent, proper counseling, agreement to medication adherence, and undetectable viral load maintained through pregnancy).”
Comfort in optimal scenario clinical vignette: US-based patient	“A pregnant patient living with HIV presents to your practice to discuss breastfeeding. She has had an undetectable viral load through pregnancy. She tells you she has read about BFHIV and plans to go forward with it. You discuss US guidelines which recommend avoiding breastfeeding.^b^ She feels she has a good understanding of the risk and benefits of BFHIV and maintains her desire and plan to breastfeed while continuing her current antiretroviral regimen. What is you comfort level in supporting this patient to breastfeed?”
Comfort in optimal scenario clinical vignette: patient immigrating from outside the US	“A PLHIV presents to your practice with her 2-week-old infant and 2 healthy children to establish care. They have just moved to the US from Malawi. The mother had regular prenatal care and had an undetectable viral load at delivery. She has been strictly breastfeeding as instructed by her obstetrician and pediatrician in Malawi prior to their move. What is your comfortable in supporting this patient to breastfeed?”

^a^ Responses based on a Likert Scale, including extremely comfortable, somewhat comfortable, neither comfortable nor uncomfortable, somewhat uncomfortable, and extremely uncomfortable.

^b^ Survey was written and distributed before March 2022 Center for Disease Control and Prevention BFHIV updates.

BFHIV, breastfeeding with HIV; PLHIV, person/people living with HIV; US, United States.

The target population was US based providers with experience in the clinical issues associated with BFHIV. The online survey was sent with one or two email reminders to multiple national breastfeeding and HIV provider listservs: ReproID 414 members, American Academy of Pediatrics Section on Breastfeeding 845 members, American Academy of HIV Medicine ∼14,500 members (member email open rate reported as 20–35%), and Pacific AIDS Education Training Center 2585 members. Total listservs membership included ∼18,344 members from different states and countries. Email open rates were not available for all distribution networks; an adjusted response rate could not be calculated. Information on overlapping membership between listservs was not obtained. All survey responses were anonymous; survey respondents were not compensated. Respondent IP addresses were reviewed for duplicates.

Community advisors were an essential part of the research team from the onset of study design. People with direct or indirect experience with BFHIV were invited to participate through flyers distributed by local providers; positive HIV status was not a requirement for the role and applicants were not asked their status. Community advisors were research collaborators who deeply informed survey content as well as interpretation and dissemination of results, and they were compensated for their time in these roles. Advisors had an option to continue in an uncompensated role regarding presentations and publications.

Survey responses occurred between June 21 and July 22, 2021. Inclusion criteria were 80% survey completion and primary practice in the US. Four researchers (G.C., H.K., A.L., and E.S.Y.) independently reviewed all responses for inclusion, assessed that each set of demographic responses was distinct, and collated free-response identified institutions to eliminate duplications. The University of California San Francisco Institutional Review Board approved this study. All participants provided informed consent.

### Quantitative data analysis

Quantitative methods included the following: descriptive statistics, Fisher's exact, Kruskal Wallis, Wilcoxon rank sum and signed rank nonparametric testing, and ordinal regression. *p* Values <0.05 were considered statistically significant. Wilcoxon rank sum or Kruskal Wallis tests were performed if there were two or multiple populations of interest (i.e., specialty, region, prior experience with infants of people BFHIV who had side effects attributed to HIV medical management or prophylaxis), respectively, to assess if there were statistically significant differences in associations between independent variables (e.g., specialty) and outcomes (four measures of comfort, Table 1). Independent variables that showed significant association were analyzed using ordinal regression to assess effect sizes. Subspecialty analysis was unable to be performed due to low sample sizes. Quantitative data were managed and analyzed with Qualtrics, Excel 365 (16.5; Microsoft Corp., Redmond, WA), and Stata (15.1; StataCorp, LLC, College Station, TX).

Assumptions for each ordinal regression model were tested. The presence of multi-collinearity was assessed with variation inflation factor (vif <4 for all variables). Proportional odds (between comfort and independent variables) were calculated (“omodel” command), except for one model that did not meet the assumptions for ordinal regression and so a partially constrained/generalized ordered logit model was utilized (“gologit2” command; Wald test of parallel lines assumption tested).

### Qualitative data analysis

Free response questions were analyzed by four researchers (G.C., H.K., A.L., and E.S.Y.) with the application of reflexive thematic analysis, a process of peer-debriefing and conceptualizing themes as described by Braun and Clarke.^24,25^ This framework facilitates the recognition of emerging patterns to explore respondent's individual experiences, perspectives, and practices. The aforementioned researchers collaboratively generated initial codes, cyclically refined them against the original data set, and grouped them into major and minor themes. Final key themes were retained based upon their frequency, richness, and alignment with the research questions.

## Results

### Demographics

The survey was completed by 146 participants; 100 respondents met the inclusion criteria (Table 2). The crude response rate was 0.8% (146 of ∼18,344 listserv members). Question sample size varied due to skipped questions (*n* = 91–100 for quantitative questions and *n* = 78–87 for qualitative questions), questions that allowed multiple answer choices (*n* = 90–108), or branching logic (*n* = 38–100). All survey responses correlated to a unique IP address and every individuals' set of demographic responses was distinct, indicating that there were no duplicate responses. Most respondents were White, female, and/or physicians. The most common clinical setting was an academic site, and the most common specialties were pediatrics and obstetrics-gynecology (Tables 2 and 3).

**Table 2.** Demographics of Respondents Meeting Inclusion Criteria

Characteristic	Quantitative,* n *(%)	Qualitative,* n *(%)
Years in practice	*n* = 99^a^	*n* = 86^a^
0–5	16 (16.2)	15 (17.2)
6–10	18 (18.2)	14 (16.1)
11–15	16 (16.2)	13 (15.0)
16–20	13 (13.1)	9 (10.4)
>20	36 (36.4)	35 (40.3)
Identity	*n* = 108^a,b^	*n* = 93^a,b^
American Indian/Alaskan Native	2 (2)	2 (2.2)
Asian	10 (10)	8 (8.6)
Black or African American	10 (10)	9 (9.7)
Hispanic or Latino	4 (4)	3 (3.2)
White	77 (77)	69 (74.1)
Other (e.g., South Asian, Middle Eastern, Asian American, choose not to disclose)	5 (5)	2 (2.2)
Gender^c^	*n* = 100	*n* = 87^a^
Female	87 (87)	75 (86.2)
Male	13 (13)	12 (13.8)
Practice setting	*n* = 100	*n* = 87^a^
Academic	72 (72)	65 (74.7)
Community	22 (22)	18 (20.7)
Private	5 (5)	3 (3.4)
Other	1 (1)	1 (1.2)
Professional role	*n* = 100	*n* = 87^a^
Advanced Practice Provider (i.e., Nurse Practitioner, Physician Assistant, midwife)	14 (14)	11 (12.6)
Pharmacist	4 (4)	4 (4.7)
Lactation Consultant	1 (1)	1 (1.2)
Registered Nurse	4 (4)	3 (3.4)
Physician	77 (77)	68 (78.1)
Specialty	*n* = 99^a^	*n* = 86^a^
Obstetrics and gynecology	13 (13.1)	13 (15.1)
Pediatrics	55 (56.6)	49 (57.0)
Family Medicine	8 (7.1)	7 (8.1)
Internal Medicine	18 (1)	12 (14.0)
Other	5 (4)	5 (5.8)
US region^d^	*n* = 100	*n* = 87^a^
West	21 (20.6)	21 (24.1)
South	28 (27.5)	22 (25.3)
Midwest	17 (16.7)	15 (17.2)
Northeast	34 (34.3)	29 (33.3)
Unique US institutions	84	

^a^ Missing data from skipped questions and choose not to disclose influences sample size.

^b^ Respondents were allowed to select multiple answers.

^c^ Options included Male, Female, Transgender Woman, Transgender Man, Other (free text), Choose not to disclose.

^d^ One non-US participant excluded from the study.

US, United States.

**Table 3.** Respondents Categorized by Subspecialty

Specialties by respondent subspecialties^a^	*n*
Obstetrics and Gynecology	
Obstetrics and Gynecology	8
Obstetrics and Gynecology—Maternal Fetal Medicine	5
Pediatrics	
Pediatrics—General Pediatrics	24
Pediatrics—Hospital Medicine	1
Pediatrics—Infectious Disease	22
Pediatrics—Neonatology	8
Internal Medicine	
Internal Medicine—General	2
Internal Medicine—Infectious Disease	16
Other	
Clinical Pharmacy	1
Internal Medicine and Pediatrics	1
Midwife	1
Virology	1
N/A	1

^a^ No family medicine subspecialties were reported by respondents.

The majority of providers were from the northeast US and in practice for >20 years. The most common reason for exclusion from the study was an incomplete survey. The excluded responses had similar predominant demographics (i.e., White, female, physicians, pediatrics, longer years in practice). An institutional protocol to support BFHIV was noted for 8 of 84 unique institutions, 10%, whereas respondents were unsure if this type of protocol existed for 7 institutions, 8%, and endorsed that this type of protocol did not exist for 69 institutions, 82%.

### Quantitative results

Of 100 included respondents, 86% had counseled PLHIV on infant feeding choices in general, while a smaller subset, 56%, endorsed counseling PLHIV more specifically about the potential to breastfeed (Table 4). When asked about not only counseling but also supporting a PLHIV during breastfeeding, one respondent had managed this entire process for over ten patients, and around one third, 31%, of respondents reported having provided care for up to three patients from the counseling to the breastfeeding phases. However, over half of respondents, 57%, had never counseled and subsequently supported a patient during BFHIV (*n* = 94).

**Table 4.** Individual Practice Regarding Provider Counseling, Supporting, and Caring for Patients Breastfeeding with HIV

Questions	Total sample size	Yes/any occurrence,* n *(%)^a^	No/never,* n *(%)^a^	Unsure,* n *(%)^a^
Have you counseled a PLHIV about their infant feeding choices (breastfeeding vs. formula feeding) in your practice? If so, how often in the last year?	*n* = 100	86 (86)	14 (14)	0 (0)
At least weekly		10 (10)		
At least monthly		11 (11)		
At least 5 times in the past year		24 (24)		
At least 5 times in the past 5 years		23 (23)		
Once or twice in my career		18 (18)		
Have you counseled a PLHIV about the potential to breastfeed in your practice? If so, how often in the last year?	*n* = 100	56 (56)	44 (44)	0 (0)
At least weekly		3 (3)		
At least monthly		5 (5)		
At least 5 times in the past year		7 (7)		
At least 5 times in the last 5 years		15 (15)		
Once or twice in my career		26 (26)		
How many PLHIV have you counseled through and subsequently supported during breastfeeding?	*n* = 94^b^	40 (42.5)	54 (57.5)	
1–3		29 (31)		
4–6		6 (6.3)		
7–10		4 (4.2)		
10 or greater		1 (1)		
Have you ever cared for a PLHIV who breastfed?	*n* = 100	42 (42)	57 (57)	1 (1)

^a^ All percentages are reflective of total sample size.

^b^ Missing data from skipped questions influences sample size.

PLHIV, person/people living with HIV.

**FIG. 1.**
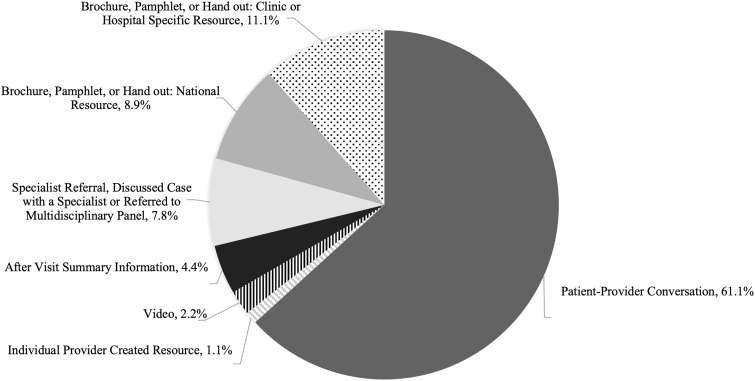
Methods of providing counseling regarding BFHIV. Question allowed for multiple responses with 90 responses from 56 respondents. BFHIV, breastfeeding with HIV.

In regard to caring for a PLHIV who breastfed during a single patient encounter, 42% of providers reported this type of limited experience (*n* = 100). The most common sources of BFHIV guidance were discussions with colleagues and literature review. Counseling was most frequently provided through direct conversation with the patient, as opposed to specialist of referral or handout (Fig. 1). Almost all, 99% (*n* = 99), of the providers strongly agreed that they should answer questions about BFHIV if a patient asks about the option, while only 72% (*n* = 100) of providers strongly agreed that providers should initiate conversations with PLHIV about breastfeeding (Fisher exact, *p* < 0.05).

Personal or institutional BFHIV experience was associated with greater provider comfort outside an optimal scenario and in optimal scenario clinical vignettes (Table 5). Institutional protocols supporting BFHIV were also associated with higher degrees of provider comfort in optimal scenario clinical vignettes. However, when respondents were asked about their general comfort in optimal or outside of optimal scenarios, institutional protocols were not associated with greater provider comfort. Responses were also evaluated by region, specialty, and other characteristics as follows. Variables not associated with provider comfort were specialty (*n* = 99), region (*n* = 100), years in practice (*n* = 99), prior experience with infants of people BFHIV who had side effects attributed to HIV medical management or prophylaxis (*n* = 38), and experience counseling a PLHIV about general infant feeding choices (*n* = 100; *p* value >0.05 with nonparametric testing; Table 5).

**Table 5.** Variables Impacting Provider Comfort with Breastfeeding with HIV in Clinical Situations

Variable*^a^* (% yes,* n*)	Having an institutional protocol (10%,* n = *100)		Providers at the institution have ever cared for a PLHIV who breastfed (41%,* n = *100)		Having personally cared for a PLHIV who breastfed in any clinical setting (42%,* n = *100)		Having counseled a PLHIV about the potential to breastfeed (56%,* n = *100)	
	OR*^b^* (95% CI)	*p*	OR (95% CI)	*p*	OR (95% CI)	*p*	OR (95% CI)	*p*
General comfort in optimal scenario^c^	0.6 (0.2–2.2)	0.43	1.8 (0.8–4.6)	0.18	1.7 (0.68–4.0)	0.27	1.7 (0.7–4.1)	0.21
General comfort outside of optimal scenario	3.1 (0.9–10.0)	0.06	3.7 (2.0–10.0)	<0.001^d^	6.2 (2.7–14.2)	<0.001^d^	4.1 (1.9–9.0)	<0.001^d^
Comfort in optimal scenario clinical vignette: US-based patient	4.9 (1.3–18.1)	0.02^d^	3.7 (2.0–10.0)	<0.001^d^	5.1 (2.3–11.3)	<0.001^d^	3.9 (1.8–8.4)	<0.001^d^
Comfort in optimal scenario clinical vignette: patient immigrating from outside the US	4.7 (1.2–19.0)	0.02^d^	3.7 (2.0–9.6)	<0.001^d^	4.8 (2.2–10.6)	<0.001^d^	2.9 (1.4–6.2)	0.005^d^

^a^ Variables that did not contribute (i.e., found to have *p* value >0.05 with non-parametric testing): specialty (i.e., pediatrics, obstetrics and gynecology, internal medicine, infectious disease, family medicine; *n* = 99), region (*n* = 100), years in practice (*n* = 99), prior experience with infants of people BFHIV who had side effects attributed to HIV medical management or prophylaxis (*n* = 38), having counseled a PLHIV about their infant feeding choices (*n* = 100).

^b^ Log OR.

^c^ Optimal scenario definition: sustained viral suppression during pregnancy and postpartum and patient-centered care.

^d^ Statistically significant, *p* < 0.05.

BFHIV, breastfeeding with HIV; CI, confidence interval; OR, odds ratio; PLHIV, person/people living with HIV; US, United States.

**FIG. 2.**
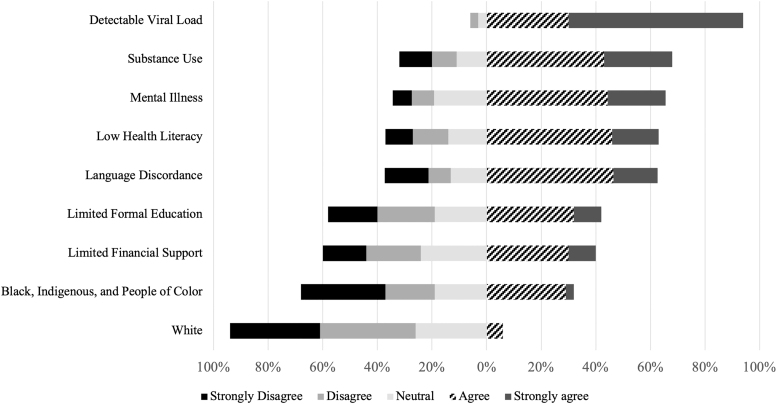
Provider perception of barriers to supporting BFHIV, by patient identity or characteristic. Level of agreement reported as categorized percentages. BFHIV, breastfeeding with HIV.

**FIG. 3.**
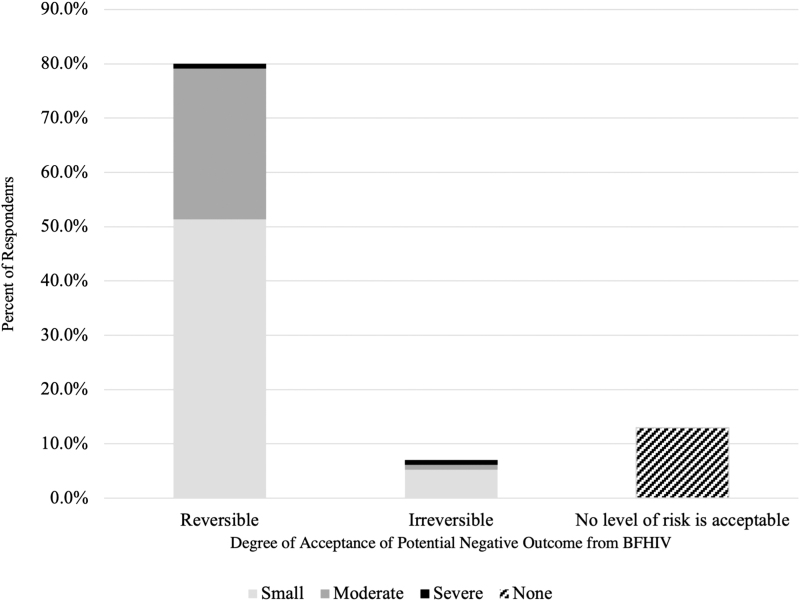
Provider perception of acceptable negative infant outcomes from BFHIV. Question allowed for multiple responses with 115 responses from 91 respondents. BFHIV, breastfeeding with HIV.

Over half of providers, 58%, felt that there were barriers in their workplace to supporting BFHIV (*n* = 100). The strongest barriers to clinicians supporting BFHIV were thought to be detectable viral load, substance use, mental illness, low health literacy, and language discordance (*n* = 100 for all barriers, except mental illness and language discordance *n* = 99, Fig. 2). Respondents perceived that White patients faced fewer barriers to BFHIV. When asked about acceptable risk to infants, 80% of providers felt a reversible negative outcome would be tolerable, while 13% endorsed that no level of risk is acceptable (*n* = 91, Fig. 3).

Respondents were divided on how they felt their institution was able to incorporate the ethical principles of beneficence, non-maleficence, and patient autonomy into BFHIV care. While 39% felt their institution did balance these principles, 35% felt unsure and 24% felt that their institution did not appropriately balance these principles (*n* = 95).

### Qualitative results

A total of 87 providers contributed 322 unique free text responses to 7 survey questions. The key themes were provider discomfort around BFHIV, patient's informed choice, and paternalism, as well as maternal and infant benefits and harms. Key themes and subthemes are exemplified with quotes most representative of the pattern of responses (Table 6).

**Table 6.** Representative Quotes of Key Themes and Subthemes

Provider discomfort	
***Personal ethics:*** “I feel the need to protect the infant and think it isn't ethical to put the infant at increased risk, therefore we have to this point have only allowed women with stable suppressed viral loads to [breastfeed] their infants.”	
***Adverse outcomes:*** “One transmission. isn't that enough?”	
***Provider disagreement:*** “Some of the providers in our small group believe that our guidelines should be liberalized … other providers feel that we should not allow BFing among WLHIV under any circumstance. It has been difficult to get consensus.”	
***Lack of guidelines or data:*** “I would not feel comfortable because there aren't specific guidelines or literature to support the care, however I'm very interested in learning more for those who are interested in breastfeeding to be able to support that decision.”	

Patient's informed choice	Paternalism
***Recognize patient autonomy:*** “I will always support and provide care for an individual who desires to BF even if it is not my recommendation. Ultimately even though it makes me uncomfortable, what's best for the patient is to remain engaged in care.”	***Persuade the patient:*** “I would likely try to persuade her to formula feed instead.”
***Reduce patient risk of transmission:*** “I would try to help her get to a place where she was as low-risk as possible to breastfeed, i.e. on meds and virally suppressed with good lactation support.”	***Compel using medical legal risk:*** “Mother with HIV (undetectable prior to and during pregnancy) … decided NOT to breastfeed because CPS was called during delivery hospitalization, and mother was fearful of legal implications.”
***Extensive counseling:*** “Provide them information so they can make an informed decision. If there is increased risk of transmission I would be sure they understood this.”	***Defer to hospital policy:*** “Our hospital policy is a strict no breastfeeding with HIV no matter the viral load or compliance with treatment.”
***Rely on provider teamwork and an interdisciplinary team:*** “Our Perinatal team reviews and discusses options and joint patient-provider decision making … express our formal recommendation.”	***Refuse to provide care:*** “Some clinical staff have refused to care for PLHIV who choose to breastfeed (even if the person fits ideal conditions). It has been very time consuming and emotionally distressing (both for providers and for PLHIV) to discuss breastfeeding as an option.”

BF, breastfeeding; CPS, Child Protective Services; PLHIV, person/people living with HIV; WLHIV, women living with HIV.

**FIG. 4.**
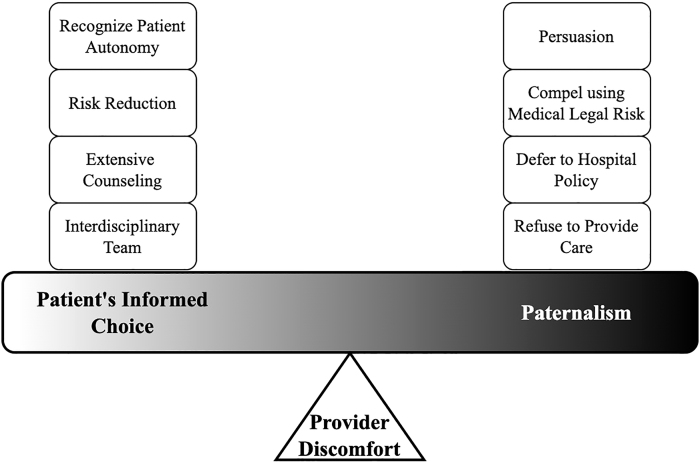
Patient care spectrum emerging from qualitative analysis. Provider discomfort as the apex of the patient care spectrum scale with subthemes represented by blocks on the scale.

Respondents expressed discomfort around BFHIV in relation to patient counseling and care, with discomfort arising from personal ethics, potential adverse outcomes, provider disagreements within and between specialties, and lack of consensus guidelines or data. Providers noted discrepancies among hospital and national guidelines, as well as insufficient data to inform BFHIV management. Providers expressed discomfort around goals to honor the role for parents in feeding choice, while protecting infants from the risk of infection. Their responses to this discomfort reflected this tension, between patient's informed choice and paternalism (Fig. 4). Providers practiced anywhere along the spectrum of these patient care styles and often borrowed from subthemes of both.

Those prioritizing patient's informed choice expressed a desire to recognize patient autonomy. A majority referred to the extensive counseling involved in supporting a patient to BFHIV and continual efforts to reduce transmission risk regardless of the provider's personal discomfort. Providers described building patient-provider trust, ensuring patient understanding of risks and benefits, further exploring the BFHIV decision, encouraging medication adherence, and increasing frequency of visits and laboratory testing. Respondents often sought counsel from other providers or teams before making recommendations, counseling, or supporting a person to BFHIV.

Providers cited paternalism at all levels of the health care system, most frequently interpersonal and institutional. Those favoring paternalism often used language of persuading the patient against BFHIV in an effort to reduce vertical transmission. Many compelled through counseling on the medical risks, advocating for exclusive formula use, or by referencing legal risk, which could involve signing an ‘against medical advice’ form, incorporating hospital risk management, and calling Child Protective Services. Often, providers took a deferential stance to their hospital policy. Occasionally, the respondent or other members of their health care team refused to care for a PLHIV who chose to breastfeed.

As providers struggled with uncertainty, many referenced their prior positive and negative clinical experiences. Providers frequently cited maternal and infant benefits and harms, and subthemes that arose were:

*Benefits:* “The benefits are numerous. Promotes maternal/infant bonding. Better immune protection for the infant. More affordable than replacement feeding. In women from immigrant communities, there is nothing that makes them stand out among peers or “outs” their HIV+ status.”

*Harms:* “Stigma from others, maternal stress or concern about passing HIV on to her child, limited support in developed nations for this practice. Moms are “shamed” at our hospital if they verbalize a desire to breastfeed their infants.”

Given the sensitive and nuanced nature of negative medical outcomes with BFHIV, providers were asked with an open-ended question about specific instances of vertical HIV transmission from breastfeeding. Of four responses documenting vertical HIV transmission, three described scenarios of detectable viral loads or inconsistent antiretroviral therapy (ART) in the periods leading up to transmission. One respondent did not provide the context of vertical transmission.

By the end of the survey, 26% of respondents noted that the survey changed their perspective on BFHIV (*n* = 78). One provider endorsed, “Yes, it made me more open to the idea,” while others maintained prior views on both ends of the practice spectrum such as, “No … because formula feeding has a non-zero chance of HIV transmission to the infant,” and, “No, I am already very pro-BFHIV and am frustrated that not more providers in the US support PLHIV to choose breast-feeding options.”

## Discussion

A significant portion of providers who responded to our survey are counseling and caring for patients BFHIV in the US. Over half of respondents in our study noted they had counseled PLHIV on the potential to breastfeed and 42% reported caring for PLHIV who breastfed, compared to 29% in a similar national survey from 2016.^11^ There has been an increasing awareness that patients in high-income countries have either breastfed with HIV^26^ or desire to.^27^ Further, published reports are likely to underestimate the prevalence of BFHIV, as patients may be reluctant to share with providers that they are breastfeeding against provider recommendations or national guidelines.^26,28^

Our results also highlight how clinical practices, responding to patient desires and values with a recognition of patient autonomy, can evolve and diverge from national guidelines that methodologically rely on evidence from clinical trials. US BFHIV guidelines have moved from strict avoidance of BFHIV in all cases^2^ to risk reduction^29^ and more supportive practices.^21^ As clinical practice adapted ahead of guideline changes, we found that providers struggled with the tension between responding to parents' choices, while simultaneously protecting infants from risk of infection and following recommendations from governing bodies.

Provider discomfort was a prominent theme that manifested in a spectrum of patient care ideologies, ranging from paternalism to patient informed choice, given the need to balance the provider's duty to protect the infant with the parent's infant feeding choices in every individual patient case. Historically provider-patient relationships were based in paternalism,^30^ in which physicians overruled a patient's preferences or choices in accordance with the physicians' perceptions of beneficence and non-maleficence.^31^

Over time, patients have been recognized as having more active roles in decisions about their care, leading to the practice of shared decision making, moving away from paternalism and toward patient's informed choice. The concept of patient's informed choice upholds the patient as the final decision maker after receiving medical information and advice, which places patients at the center of their care and recognizes current and future lived experiences. This framework acknowledges that patients may have values and preferences informing their decisions, and these may differ from those of medical providers and public health professionals.^32^

In scenarios with a very low risk of HIV transmission, many experts in the US and Canada believe that infant feeding choices belong entirely to the PLHIV and that the health care provider has a responsibility to serve as an advisor and educator.^5,33^ They have pointed out that requiring patient risk to equate to zero is “at odds with the autonomy of parents living with HIV and their fundamental right to make informed choices about their children's care.”^5^

Gross et al also maintain that the maternal and infant benefits from BFHIV in cases with sustained undetectable viral loads can outweigh the right of the physician or institution to act as the sole decision maker in a paternalistic manner, and the authors propose that patient autonomy should be upheld in applicable cases.^16^ Our study provides evidence that there is a wide range of BFHIV risk tolerance at the institutional and interpersonal levels, which can limit patient autonomy as providers may impose their personal ethics and risk tolerance on their patients.

Reliance on physicians' perceptions in patient care can exacerbate structural inequity.^34–36^ HIV disproportionately impacts Black people in the US,^19^ and BFHIV patient populations in high-income countries include many Black immigrants.^37^ Given the degree to which Black communities are affected by HIV in high-income countries and that both maternal^38^ and infant^39^ mortality are higher for Black patients in the US, it is notable that provider responses in our study overwhelmingly felt neutral or disagreed that a patient identifying as White was a barrier, whereas almost one third of respondents considered a patient identifying as Black, Indigenous, or a Person of Color to be a barrier to supporting BFHIV. These findings underscore the importance of identifying and dismantling social causes of health disparities, including internalized interpersonal and structural racism, implicit bias, poverty, xenophobia, and lack of access to health care.

Most providers in this study reported some degree of risk tolerance with BFHIV, particularly for potential reversible negative outcomes. However, a notable small number of respondents endorsed that no level of risk is acceptable. In contrast, Kahlert et al argue that it is imperative to inform PLHIV about the potential to breastfeed when the overall risk is low (i.e., optimal scenario).^20^ Despite emerging perspectives advocating for discussion of BFHIV in optimal scenarios,^10,16,20,28^ this study found that some US providers remained uncomfortable supporting BFHIV due to interprovider disagreement, potential adverse outcomes, and lack of data or guidelines. Absence of BFHIV consensus nationally may contribute to provider confusion^1^ and reinforce reliance on provider personal ethics or risk tolerance.

There were several limitations to this study, including respondent demographics, response rate, and sample size. Our demographics were skewed toward White, female, physicians, pediatrics, and academic sites. We distributed our survey using listservs most likely to reach medical professionals who have experience with BFHIV, which included general pediatricians or HIV-related providers. Thus, our results cannot be generalized to all providers who support breastfeeding, such as obstetricians without HIV focus. As this study assessed provider perceptions, our data do not represent the patient voice or depict patient perspectives on BFHIV considerations. We also had a small sample size and a low response rate, despite high listserv membership, but we anticipate our online survey was not opened by all listserv members, particularly given that the email open rate of our largest distribution network was a fraction of the total reported membership.

Further, our sample size was not large enough to evaluate answers by medical subspecialty (e.g., Neonatology, Maternal-Fetal Medicine), presenting an opportunity for future study. Subsequent studies should additionally address the challenges of counseling adolescent parents living with HIV about feeding choice, given they are at increased risk for disengaging from care.^40^ Moreover, the authors recognize that transgender men and gender nonbinary individuals may be birthing parents, and may choose to feed at the chest.^41,42^ Unfortunately, because the term “breastfeeding” was utilized in our survey, the results cannot necessarily be extrapolated to apply to chestfeeding. We advocate for the incorporation of chestfeeding in future studies to be inclusive of all families.

This study highlights the challenging tension faced by US providers around counseling and supporting infant feeding choice for PLHIV. Additional studies will ideally identify markers or interventions that help providers and parents work together to reduce the risk of vertical transmission from breastfeeding to closer to zero. Effective counseling must not only provide accurate and accessible information about risk of perinatal HIV transmission from breastfeeding but also acknowledge that parents will ultimately choose their infant feeding method weighing the risks and benefits in different ways based on their own preferences and values. Medical providers and institutions face a challenging yet essential mandate to strive to optimize patient health outcomes in a way that upholds patients' autonomy, advances equitable access to infant feeding choice, and dispels stigma. National and global guidelines can and should play a proactive role in helping providers navigate this challenge, as the landscape of risk changes.
